# In vitro evaluation of physiologically relevant concentrations of teriflunomide on activation and proliferation of primary rodent microglia

**DOI:** 10.1186/s12974-016-0715-3

**Published:** 2016-09-22

**Authors:** Tanja Wostradowski, Chittappen Kandiyil Prajeeth, Viktoria Gudi, Jessica Kronenberg, Sina Witte, Marina Brieskorn, Martin Stangel

**Affiliations:** 1 Clinical Neuroimmunology and Neurochemistry, Department of Neurology, Hannover Medical School, Carl-Neuberg-Str.1, 30625 Hannover, Germany; 2 Center for Systems Neuroscience, University of Veterinary Medicine Hannover, 30559 Hannover, Germany

**Keywords:** Multiple sclerosis, Teriflunomide, Microglia, Proliferation, Pro-inflammatory (M1), Anti-inflammatory (M2)

## Abstract

**Background:**

Teriflunomide, an inhibitor of dihydroorotate dehydrogenase, is thought to ameliorate multiple sclerosis by reducing activation-induced proliferation of lymphocytes, which is highly dependent on de novo pyrimidine synthesis. Nevertheless, its immunomodulatory effects on resident glial cells in the central nervous system are only poorly understood.

**Methods:**

In this study, we employed physiologically relevant concentrations of teriflunomide and investigated its effects on survival, proliferation, activation, and function of primary rat microglia in vitro.

**Results:**

We demonstrate that teriflunomide had no cytotoxic effect on microglia and had only a minor impact on microglial activation. In a concentration- and time-dependent manner, teriflunomide significantly downregulated surface expression of the co-stimulatory molecule CD86. Furthermore, in the highest concentration applied (5 μM), it slightly increased the expression of interleukin-10 in microglia in response to lipopolysaccharide. Treatment with low concentrations of teriflunomide (0.25–1 μM) did not have any impact on the activation or proliferation of microglia. At 5 μM concentration of teriflunomide, we observed a reduction of approximately 30 % in proliferation of microglia in mixed glial cell cultures.

**Conclusions:**

Taken together, our in vitro findings suggest that at higher concentrations, teriflunomide potentially exerts its effects by reducing microglial proliferation and not by modulating the M1-/M2-like cell differentiation of primary rat microglia. Thus, teriflunomide has no major impact on the plasticity of microglia; however, the anti-proliferative and minimal anti-inflammatory effects might be clinically relevant for immune modulation in the treatment of neuroinflammatory CNS diseases such as multiple sclerosis.

**Electronic supplementary material:**

The online version of this article (doi:10.1186/s12974-016-0715-3) contains supplementary material, which is available to authorized users.

## Background

Teriflunomide is an immunomodulatory disease-modifying therapy (DMT) for relapsing-remitting multiple sclerosis (RRMS). It is thought to act primarily via a specific, non-competitive, and reversible inhibition of the activity of the mitochondrial enzyme dihydroorotate dehydrogenase (DHODH), which is required for de novo pyrimidine synthesis of rapidly dividing cells such as activated lymphocytes [1, 2]. Resting lymphocytes synthesize the nucleotide pyrimidine through the DHODH-independent salvage pathway and therefore are largely unaffected by teriflunomide [3]. Teriflunomide is the active metabolite of leflunomide in vivo which is an effective agent in the treatment of rheumatoid arthritis (RA) [4].

Several studies indicate that teriflunomide rather exerts cytostatic and not cytotoxic effects on actively proliferating lymphocytes in the periphery [5, 6]. This limits the availability of autoreactive T and B cells that can infiltrate into the central nervous system (CNS) in CNS inflammatory diseases such as multiple sclerosis (MS) [7]. Other effects of teriflunomide such as the inhibition of adhesion molecules, cytokines, protein tyrosine kinases, nuclear factor-kB (NF-kB) activation, and cyclooxygenase 2 activity have also been demonstrated in some in vitro studies, suggesting that teriflunomide in addition to its anti-proliferative effects may also impact signal transduction, migration, and inflammatory processes [8, 9].

Pharmacokinetic analysis of patients treated with teriflunomide revealed a concentration of 20–60 mg/l in the serum. Owing to its low permeability through the blood-brain barrier (BBB), only approximately 1–2 % of the serum concentration of drug reaches the CNS, which is approximate to a concentration of 1–3 μM in vivo [10, 11]. Thus, CNS resident cells like microglia are exposed to much lower concentrations of teriflunomide than the immune cells in the periphery. However, these concentrations are still in the range where, e.g., the DHODH is inhibited and thus therapeutic concentrations of teriflunomide may modulate microglia. Microglia are considered to play key regulatory and effector functions during the onset and progression of CNS diseases [12]. Microglial activation in the CNS during neuroinflammation can have both beneficial and detrimental effects. By virtue of their phagocytic ability, they assist in clearing debris which in turn might hasten the repair processes [13]. Furthermore, several inflammatory factors produced by activated microglia are essential for repair processes [14]. On the other hand, activated microglia serve as antigen-presenting cells and are involved in the reactivation of the CNS-infiltrating autoreactive T lymphocytes and thereby promoting neural tissue damage. Additionally, neuroinflammation triggers the release of toxic reactive oxygen species such as nitric oxide by microglia, which is highly toxic to neurons.

In order to explore the potential of teriflunomide to modulate microglia, we applied physiologically relevant concentration of 0.25–5 μM on primary rat microglia and investigated various functions.

## Methods

### Mixed glial cell cultures

Primary microglia from neonatal Sprague-Dawley rats (Crl:CD) were isolated by the method of Giulian and Baker [15] as previously described [16]. Briefly, brains were freed from meninges and dissociated mechanically and enzymatically with 0.1 % trypsin (Biochrom, Merck Millipore, Darmstadt, Germany) and 0.25 % DNase (Roche Diagnostic GmbH, Mannheim, Germany). Cells were plated at 1–2 brains per poly-l-lysine (Sigma-Aldrich, Munich, Germany) coated culture flasks (25 cm^2^; 75 cm^2^; Sarstedt, Nümbrecht, Germany) and cultivated in Dulbecco’s Modified Eagle Medium (DMEM; life technologies, Carlsbad, USA) supplemented with 10 % fetal calf serum (FCS; Biochrom, Merck Millipore, Darmstadt, Germany) and 1 % penicillin/streptomycin (life technologies, Carlsbad, USA; culture medium referred to as: MGP^+^), where indicated culture flasks were treated with granulocyte macrophage-colony stimulating factor (GM-CSF; 5 ng/ml; Peprotech; Hamburg, Germany). After 7–8 days, loosely attached microglia were harvested from mixed glial cell cultures by shaking for 30–45 min on an orbital shaker-incubator (Edmund Bühler, Hechingen, Germany) at 37 °C. Cells in the supernatant were re-plated at 3–6 × 10^4^ cells per 12-mm glass coverslip in 24-well plates for immunocytochemistry staining, directly resuspended in 500 μl MGP^+^ for BrdU flow cytometry, 5 × 10^5^ per well in 6-well plates for reverse transcription polymerase chain reaction (RT-PCR), or 2.5 × 10^5^ in 12-well plates (all Nunc, Life Technologies, Paisley, UK) for phagocytosis and flow cytometry analysis. The remaining astrocytes were washed with PBS and harvested with 0.25 % trypsin/0.05 % EDTA. After isolation, the astrocytes were incubated with an APC-conjugated mouse/rat anti-GLAST (ACSA-1) antibody and with FITC-conjugated anti-BrdU antibody.

Microglia were incubated overnight at 37 °C, 5 % CO_2_, and the following day, cells were treated with 0, 0.25, 0.5, 1, and 5 μM of teriflunomide (stock: 10 mM, dissolved in dimethyl sulfoxide (DMSO); Genzyme, Waltham, MA; USA) with or without indicated stimuli for the designated period of time for each experiment. Cell culture purity and cell viability were analyzed by immunofluorescence stainings with CD11b/c or Iba-1, markers for microglia/macrophages, and DAPI or PI, markers for cell viability. Results were quantified by flow cytometry or fluorescence microscopy.

### BrdU flow cytometry analysis of primary mixed glial cell cultures

The incorporation of 5-bromo-2′-deoxyuridine (BrdU) was analyzed in mixed glial cell cultures using flow cytometry and labeling with a conjugate anti-BrdU antibody (FITC BrdU flow kit, BD Pharmingen, CA, USA). Mixed glial cell cultures were incubated with 0.25–5 μM teriflunomide and co-treated with GM-CSF (5 ng/ml; Peprotech, Hamburg, Germany) from day 5 after preparation. The next day, 10 μM BrdU (FITC BrdU flow kit, BD Pharmingen) was added for 16 h.

After microglia isolation, the Fc receptors were blocked with mouse anti-rat CD32 for 30 min on ice (clone: D34-485; BD Pharmingen). Surface staining of microglia was done with allophycocyanin (APC)-conjugated rat anti-CD11b/c antibody for 30 min at 4 °C. Cells were then fixed and permeabilized with fixation/permeabilization buffer (Foxp3 Staining Buffer Set; eBioscience) according to the manufacturer’s instructions. The samples were treated with DNase to expose incorporated BrdU (diluted to 300 μg/ml; BrdU flow kit, BD Pharmingen) for 1 h at 37 °C and finally stained with fluorescein isothiocyanate (FITC)-conjugated anti-BrdU antibody (30 min at RT, 1:100; BrdU flow kit, BD Pharmingen). The analysis was carried out using a FACSCalibur with CellQuest software (BD). Measurements were performed in duplicates per condition and in four independent experiments.

### T cell proliferation assay

Single-cell suspensions from spleens of adult Sprague-Dawley rats (Crl:CD) were prepared in complete Iscove’s Modified Dulbecco’s Medium (IMDM) medium. After passage through a 100-μm-mesh-size cell strainer (BD Falcon), red blood cells were lysed by NH_4_Cl treatment. CD4^+^ T cells were purified by MACS technology using MagCellect rat CD4^+^ T Cell Isolation Kit (R&D Systems, Minneapolis, USA) according to the manufacturer’s instructions. The final depleted cell fraction contained the desired highly enriched CD4^+^ T cells. Purity of the enriched CD4^+^ T cells was analyzed with a phycoerythrin (PE)-conjugated anti-rat CD4 antibody (W3/25) and a FITC-conjugated anti-rat T cell receptor α/β antibody (R73). Enriched CD4^+^ T cells were labeled with 2.5 μM carboxyfluorescein succinimidyl ester (CFSE; Invitrogen, Darmstadt, Germany), respectively, for 10 min at 37 °C. Labeling of cells was stopped by the addition of ice-cold complete IMDM medium and by three washing steps. CFSE-labeled and unlabeled CD4^+^ Rat T cells (1 × 10^5^ cells/well) were seeded in 96-well microtiter plates (Nunc™ 96-Well Polystyrene Round Bottom Microwell Plates, Thermo Fisher Scientific, USA) in 100 μl medium with the addition of different concentrations of teriflunomide and stimulated with 3 μg/ml of plate-bound anti-CD3 mAb/anti-CD28 mAb. The final culture volume was adjusted to 200 μl per well. Cells were maintained at 37 °C in a humidified atmosphere with 5 % CO_2_. After 65–72 h incubation, cells were washed in PBS, collected and the absolute amount of proliferating rat T cells was determined by flow cytometry.

### RNA isolation and reverse transcription polymerase chain reaction 

To determine the messenger RNA (mRNA) levels of pro-inflammatory factors such as iNOS, TNF-α, and IL-1β, anti-inflammatory factors such as Arg1 and IL-10, and growth factor IGF-1, or mRNA level of DHODH, real-time PCR analysis was performed (Table 1).

**Table 1:** Primer used for polymerase chain reaction

Gene	Gene expression assay number
DHODH	Rn01432611_m1
IL-1β	Rn00580432_m1
IL-10	Rn00563409_m1
iNOS	Rn00561646_m1
Arg1	Rn00691090_m1
TNF-α	Rn99999017_m1
IGF-1	Rn00710306_m1
HPRT	Rn01527840_m1

*DHODH* dihydroorotate dehydrogenase, *IL-1β* interleukin-1beta, *IL-10* interleukin-10, *iNOS* inducible nitric oxide synthase, *Arg1* arginase1, *TNF-α* tumor necrosis factor-alpha, *IGF-1* insulin-like growth factor-1, *HPRT* hypoxanthine-guanine phosphoribosyl-transferase

For cytokine analysis, total RNA was isolated from microglia pre-treated with different concentrations of teriflunomide (0.25–5 μM) for 12 h and cultured for an additional 12 h with different supplements: (1) recombinant rat interferon (IFN)-γ (50 ng/ml; Peprotech, Hamburg, Germany) plus lipopolysaccharide (LPS; 100 ng/ml; Sigma-Aldrich, Munich, Germany) and (2) recombinant rat IL-4 (20 ng/ml; Peprotech, Hamburg, Germany) to induce a M1- and M2-like phenotype, respectively.

For DHODH expression analysis, we used two experimental designs. Firstly, we isolated total RNA from microglia pre-treated with different concentrations of teriflunomide (0.25–5 μM) for 12 h and cultured for an additional 12 h with medium containing IFN-γ (50 ng/ml; Peprotech, Hamburg, Germany) plus LPS (100 ng/ml; Sigma-Aldrich, Munich, Germany) or MGP+ medium. Secondly, we isolated total RNA from microglia from mixed glial cell cultures co-treated with different concentrations of teriflunomide (0.25–5 μM) and 5 ng/ml GM-CSF or medium for 12 h.

The RNeasy®Micro Kit (Qiagen, Hilden, Germany) was used according to the manufacturer’s instructions. The RNA concentration was measured using a NanoDrop 2000 spectrophotometer (Thermo Fisher Scientific, MA, USA). Complementary deoxyribonucleic acid (cDNA) was synthesized using the High Capacity cDNA Reverse Transcription Kit (Applied Biosystems, Foster City, CA, USA). qPCR analysis was performed using the StepOne™ Real-Time PCR System and appropriate TaqMan assay (Applied Biosystems; see Table 1). The experiments were performed with cDNA from 400 ng total RNA, and all primers were exon-spanning. The ΔΔCT method was used to determine differences in the expression between controls and drug-treated microglia exposed to M1/M2 or GM-CSF stimuli. Changes in the mRNA expression levels were quantified against the housekeeping gene hypoxanthine-guanine phosphoribosyl-transferase (HPRT) 1 compared with control.

### Flow cytometry

LPS (100 ng/ml; Sigma-Aldrich, Munich, Germany) stimulated microglia were cultured in the absence or presence of teriflunomide (0.25–5 μM) for 48 or 72 h. After incubation, cells were washed and harvested by 0.05 % trypsin/EDTA treatment and collected in polystyrene tubes. After centrifugation (352×*g*, 10 min), cells were resuspended in 100 μl of PBS and subsequently incubated with the fluorescent-labeled antibodies for 30 min on ice: APC-conjugated anti-rat CD11b/c mAb (OX-42, 1:100), PE-conjugated anti-rat CD86 mAb (24 F, 1:100), or appropriate isotype control for anti-rat CD86 (mouse IgG1, k, 1:100) (all from BioLegend, California, USA).

After incubation, cells were washed, pelleted by centrifugation (352×*g*, 10 min), and resuspended in 250 μl of PBS. Cells were immediately analyzed with FACSCalibur (Becton-Dickinson, San Jose, CA, USA) and CellQuest software (BD Biosciences). Dead cells were excluded by propidium iodide staining.

### Western blot analysis

For Western blot analysis, microglia were treated with or without 5 μM teriflunomide for 12 h and then stimulated additionally with LPS/IFN-γ (100 ng/ml; Sigma-Aldrich, Munich, Germany; 50 ng/ml; Peprotech, Hamburg, Germany) for 0, 15, 30, or 60 min at 37 °C. Cells were washed with cold PBS and lysed in RIPA buffer supplemented with 1 % protease and phosphatase inhibitor (Cell Signaling Technology; Roche). The protein content was measured using a Pierce BCA Protein Assay Kit (Thermo Fisher Scientific). The lysate (30 μg of protein) was mixed with Laemmli buffer and boiled for 5 min at 95 °C. After the NF-kB activation reaction, Western blot analysis was performed as described [17] using anti-total IkBα (1:1000, rabbit, Cell Signaling Technology) and β-actin (1:3000, mouse, Santa Cruz Biotechnology) followed by horseradish peroxidase (HRP)-coupled goat anti-mouse or goat anti-rabbit secondary antibodies (Santa Cruz Biotechnology).

Proteins were visualized by enhanced chemiluminescence (ECL, Pierce Biotechnology, Illinois, USA; Millipore, Massachusetts, USA) after treatment with the secondary antibody using the ChemoCam system (Intas, Science Imaging Instruments GmbH, Göttingen, Germany) according to the manufacturer’s instructions. Quantification of protein levels by densitometry was conducted on acquired images using LabImage 1D software (Kapelan Bio-Imaging Solutions, Leipzig, Germany).

### Phagocytosis assay

Phagocytic activity of microglia was determined by measuring the uptake of fluorescent latex beads on flow cytometry as previously described [18]. After 24 h teriflunomide (±12 h LPS; 100 ng/ml; Sigma-Aldrich, Munich, Germany) treatment, FITC-labeled latex beads (1 μm, Fluoresbrite™ Yellow Green carboxylate microspheres; Polysciences, Warrington, USA) were added at a cell:bead ratio of 1:100 and incubated for 1 h at 37 °C. In parallel, cells incubated with beads on ice (4 °C) served as negative controls. Non-phagocytosed and surface bound beads were removed by washing six times with ice-cold PBS. Adherent microglia were then harvested by 0.05 % trypsin/EDTA treatment, and the uptake of the beads was determined by flow cytometry (FACSCalibur; Becton-Dickinson, San Jose, CA, USA). Dead cells were excluded by propidium iodide staining.

A shift in mean fluorescence intensity (MFI) resulting from the uptake of fluorescent beads and the percentage of gated microglia that phagocytosed latex beads were used as a measure to assess phagocytosis. Active phagocytosis was then calculated by subtracting measured values of cells incubated at 4 °C from the values obtained at 37 °C.

### Statistical analysis

All experiments were performed at least four times, and arithmetic means ± standard deviation (SD) was calculated using GraphPad Prism 5.0 software. Comparison of two samples was performed using a one-sample *t* test or a paired Student’s *t* test followed by the correction for multiple comparisons by Benjamini and Hochberg [19]. Multiple samples were evaluated using repeated measures ANOVA with Bonferroni’s multiple comparison *t* tests. *P* values <0.05 were considered statistically significant (*p < 0.05, **p < 0.01, ***p < 0.001).

## Results

### Regulation of DHODH-mRNA expression in microglia

**Fig. 1:**
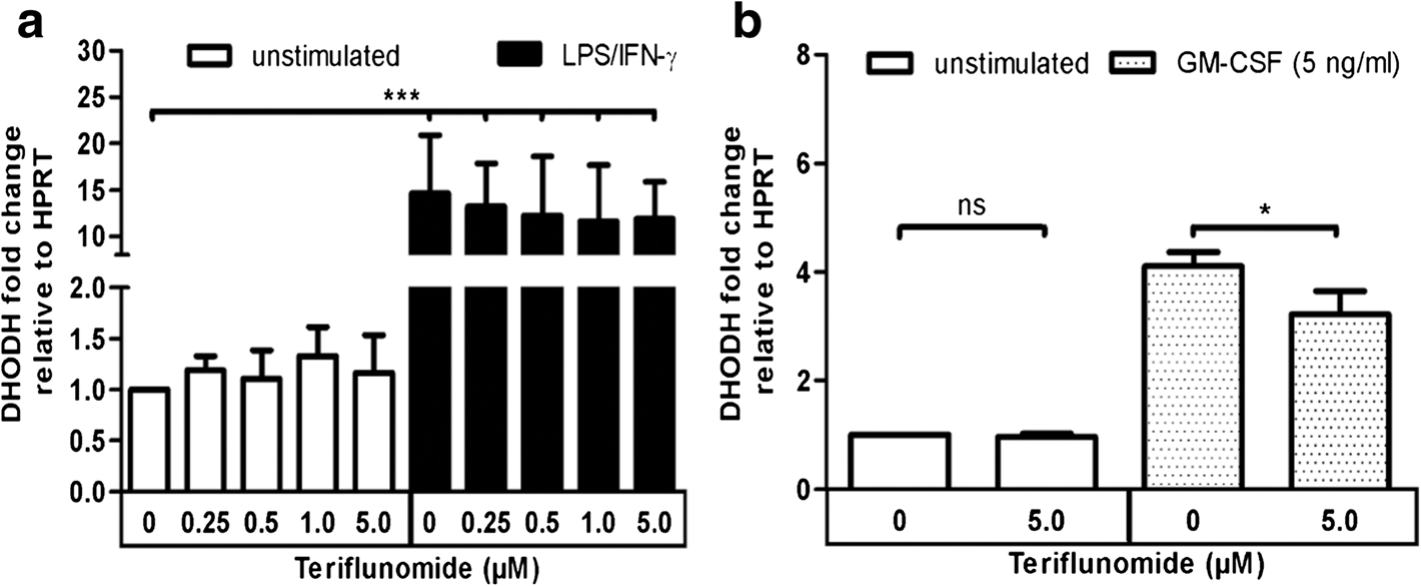
Dihydroorotate dehydrogenase (DHODH) is upregulated in activated primary microglia. Evaluation of DHODH-mRNA expression by RT-PCR in primary rodent microglia (**a**) with teriflunomide pre-treatment for 12 h following 12 h stimulation with 100 ng/ml LPS/50 ng/ml IFN-γ (*black column*) or medium (*white column*) and (**b**) teriflunomide co-treatment for 12 h with 5 ng/ml GM-CSF (*dotted column*) or medium (*white column*). Data represent four biological replicates as mean ± SD. Results are presented as the fold-change normalized to the expression of the reference gene *Hprt1* and were calculated relative to unstimulated, untreated cells. Statistical analysis was performed using repeated measures ANOVA followed by Bonferroni’s post hoc tests. *P* values <0.05 were considered significant. *Asterisks* denote a significant difference versus the indicated control (*p < 0.05)

Previous reports have revealed that teriflunomide inhibits activation-induced proliferation of T cells by targeting DHODH, which is a crucial enzyme involved in de novo synthesis of pyrimidine nucleotides [1, 2]. Therefore, we initially assessed if DHODH is induced in microglia upon activation. Following the stimulation of microglia with LPS/IFN-γ or GM-CSF, we studied the expression of DHODH-mRNA by RT-PCR. Compared to medium-treated control, we observed a significant upregulation of DHODH in LPS/IFN-γ (Fig. 1a) and GM-CSF-treated microglia (Fig. 1b). Furthermore, we observed that pre-treatment of microglia with teriflunomide prior to activation per se did not influence the mRNA expression, confirming that teriflunomide inhibition of DHODH might be at the protein level and not at the gene expression level. The slight decrease of DHODH-mRNA of approximately 20 % in GM-CSF-treated microglia by 5 μM teriflunomide was not biologically significant (Fig. 1b).

### Teriflunomide does not interfere with morphological shaping of microglia

**Fig. 2:**
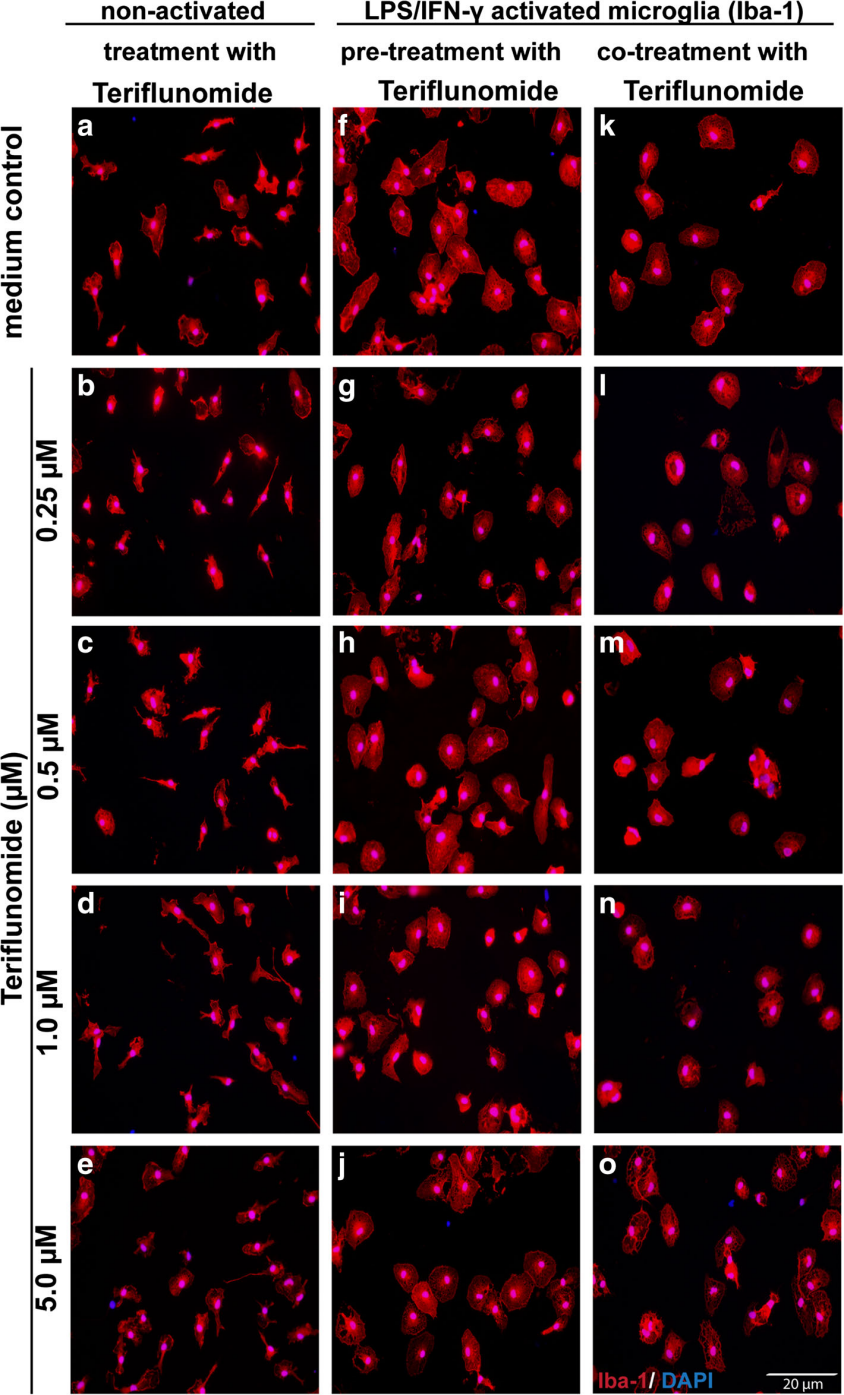
Teriflunomide does not modulate morphological shaping of non-activated and activated cultured microglia. Cultured microglia were treated with teriflunomide (0.25–5 μM) for 24 h without (**a**–**e**) or with 100 ng/ml LPS/50 ng/ml IFN-γ (pre-treatment: **f**–**j**; co-treatment: **k**–**o**). Immunofluorescence microscopy images show Iba-1^+^ microglia stained with DAPI as marker for cell viability (*n* = 4). LPS/IFN-γ-treatment (**f**–**o**) induces morphological changes in comparison with non-activated cells (**a**–**e**). *Scale bar* = 20 μm

Morphological rearrangement is a major feature during the transition of different functional stages of microglia. Thus, we investigated if teriflunomide affects the shaping of non-activated and activated microglia. After resting overnight, microglia were cultured for 24 h in the presence of teriflunomide with or without LPS/IFN-γ to induce activation in vitro. Using immunocytochemistry, we observed a morphological change of their phenotype in the presence of LPS/IFN-γ. After plating, microglia had an amoeboid appearance that was maintained after activation (Fig. 2). Teriflunomide treatment did not interfere with morphological changes of microglia when compared with non-activated and activated microglia in control medium. Except for cell culture-associated death, we did not observe any cytotoxic effects of teriflunomide (Additional file 1: Figure S2, Additional file 2: Figure S3).

### Teriflunomide reduces proliferation of primary microglia

It is known that teriflunomide inhibits active T cell proliferation; therefore, we tested if the physiologically relevant concentrations (0.25–5 μM) chosen for this study were sufficient to inhibit T cell proliferation. For this purpose, CFSE-labeled CD4^+^ T cells isolated from the spleen of Sprague-Dawley rats were stimulated on anti-CD3/anti-CD28 coated culture plates in the presence or absence of different concentrations of teriflunomide (0.25–5 μM) and CFSE dilution as an indicator of proliferation was measured by flow cytometry. Even at a concentration as low as 0.25 μM, teriflunomide significantly inhibited proliferation of T cells with a concentration-dependent effect with a maximum at 5 μM (Additional file 3: Figure S1). Having confirmed that low concentrations of teriflunomide were sufficient to mediate anti-proliferative effects in the periphery, we next investigated microglial proliferation.

**Fig. 3:**
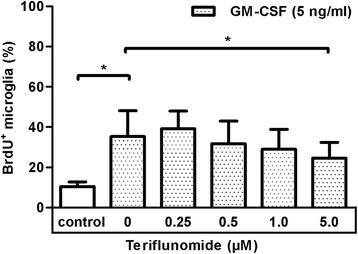
Teriflunomide influences GM-CSF-mediated microglial proliferation in vitro. Microglia were treated without (control) or with GM-CSF (5 ng/ml) in mixed glial cell cultures for 48 h. Cultures were incubated with 10 μM BrdU for the last 16 h, and dividing microglia were then visualized by labeling with a FITC-conjugated anti-BrdU antibody. For comparison of untreated (0 μM) and teriflunomide-treated (0.25–5 μM) samples (*n* = 4), a paired *t* test was used followed by correction for multiple comparisons by Benjamini and Hochberg. *P* values <0.05 were considered significant. *Asterisks* denote a significant difference versus the indicated control (*p < 0.05)

We stimulated mixed glial cell cultures containing astrocytes, microglia, and oligodendrocyte precursor cells with GM-CSF in the presence or absence of teriflunomide and assessed the proliferation and yield of microglia. While the cultures were treated with GM-CSF (5 ng/ml) and 0.25–5 μM teriflunomide on day 5, the controls received media changes only. The proliferation was determined after day 7 as described in the “Methods” section. We observed an increase in microglial proliferation in mixed glial cell cultures after treatment with 5 ng/ml GM-CSF (Fig. 3). We also detected a slight but significant reduction by 29.7 % ± 2.3 % (mean ± SD; *p* = 0.0242) in the percentage of BrdU^+^CD11b/c^+^ glial cells in GM-CSF-treated cultures after the treatment with 5 μM teriflunomide (Fig. 3). Thus, higher concentrations of teriflunomide may have an anti-proliferative effect on GM-CSF-induced proliferation of microglia. We also investigated if the drug teriflunomide could affect the proliferation of astrocytes and in this way would indirectly influence the microglial proliferation. As presented in Additional file 4: Figure S4, teriflunomide did not alter astrocytic proliferation and the expression of chosen astrocyte-secreted factors.

### Teriflunomide has no impact on microglial plasticity

Similar to macrophages, microglia can be functionally classified into either pro-inflammatory (M1-like) or anti-inflammatory (M2-like) cells [20]. We thus investigated the effect of teriflunomide on these functions. Primary rat microglia were driven into either M1- or M2-like cells by culturing them in medium containing either LPS/IFN-γ or IL-4, respectively.

**Fig. 4:**
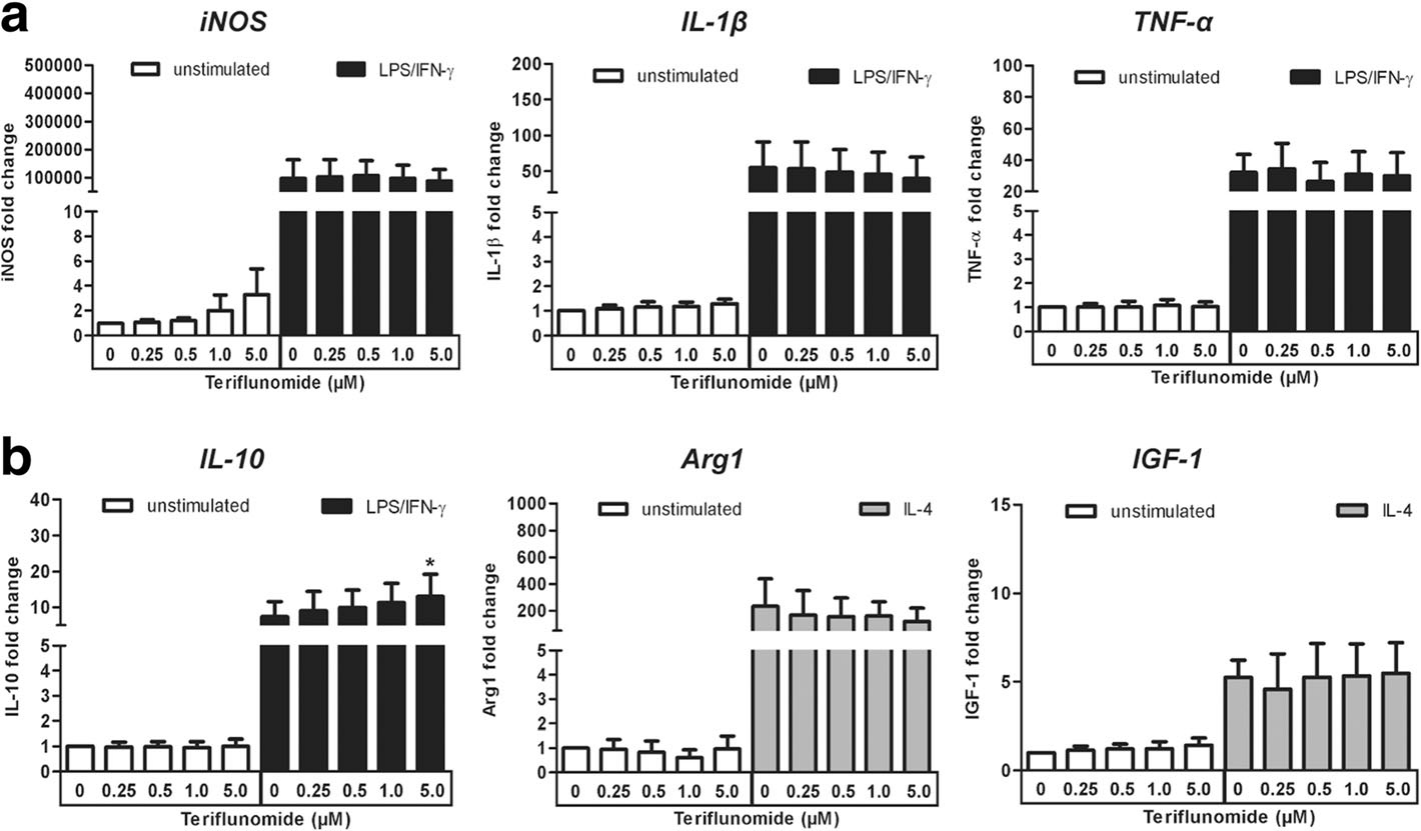
Effect of teriflunomide on the expression of pro- or anti-inflammatory mediators and growth factors in primary microglia. Expression of **a** pro- and **b** anti-inflammatory mediators in microglia with or without 12 h pre-treatment of teriflunomide (0.25–5 μM) followed by 12 h stimulation with 100 ng/ml LPS/50 ng/ml IFN-γ (*black column*), 20 ng/ml IL-4 (*gray column*), or medium (*white column*). Data represent five to six biological replicates as mean ± SD. Results were presented as the fold-change normalized to the expression of the reference gene *Hprt1* and were calculated relative to unstimulated and untreated cells. Statistical analysis was performed using repeated measures ANOVA followed by Bonferroni’s post hoc tests. *P* values <0.05 were considered significant. *Asterisks* denote a significant difference versus the stimulated and untreated control (0 μM teriflunomide; *p < 0.05)

As demonstrated in Fig. 4, pro-inflammatory factors such as iNOS, IL-1β, and TNF-α were highly upregulated in microglia treated with LPS/IFN-γ and anti-inflammatory factors such as Arg1 and IGF-1 were significantly upregulated in IL-4-treated cells. Pre-treatment of microglia with teriflunomide for 12 h prior to M1- and M2-like differentiation had no effect on the mRNA expression of any of the abovementioned pro- or anti-inflammatory genes except for a slight but significant increase in IL-10 expression in LPS/IFN-γ-treated microglia pre-treated with 5 μM teriflunomide. In summary, teriflunomide had no major impact on the plasticity of microglia to differentiate into various phenotypes.

### Teriflunomide may downregulate LPS-induced CD86 expression

**Fig. 5:**
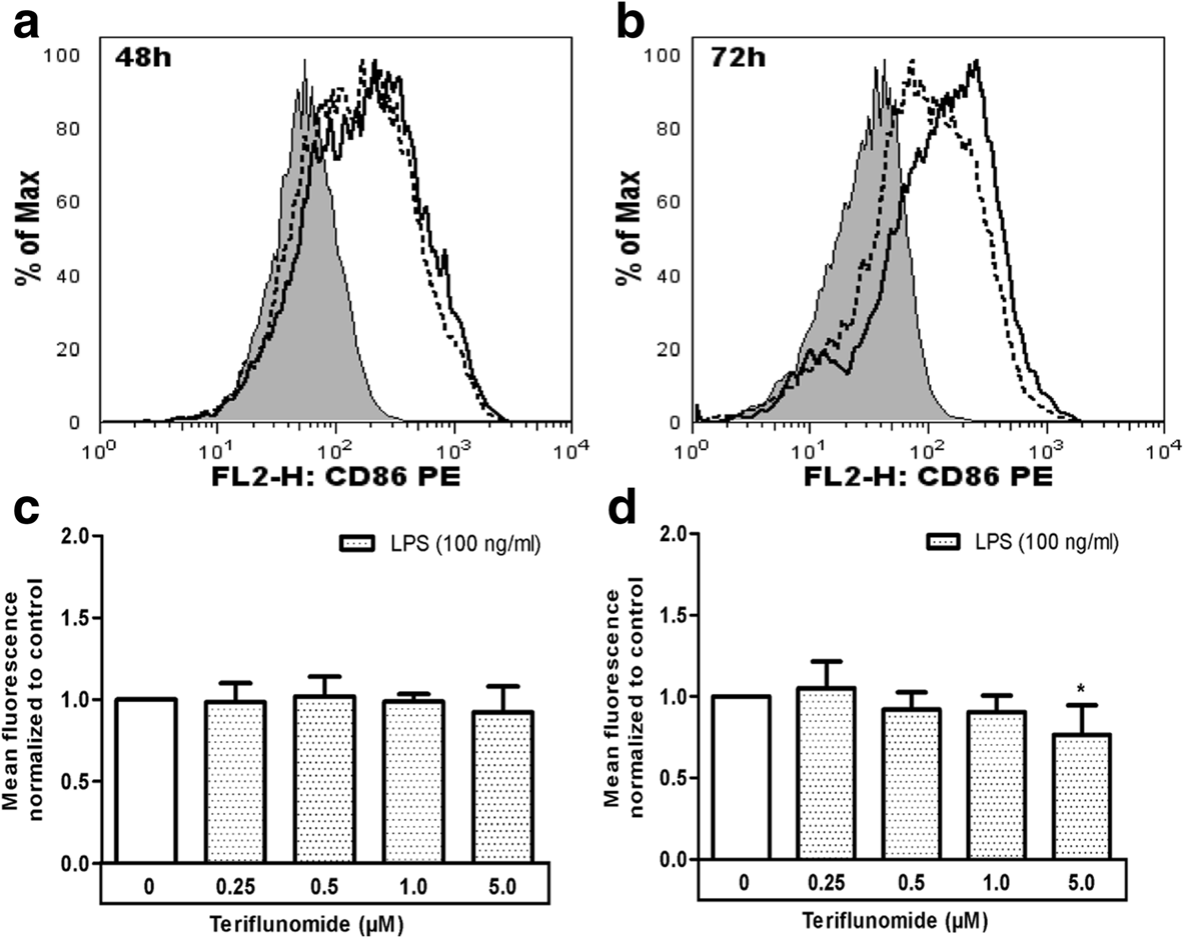
Teriflunomide leads to a decreased expression of CD86 in cultured microglia. Isolated microglia were treated with LPS (100 ng/ml) and different concentrations of teriflunomide (0.25–5 μM) for 48 or 72 h. **a**, **b** The histograms represent the CD86 expression levels on the microglial surface (*filled*: unstimulated cells; *solid line*: LPS-treated cells; *dotted line*: LPS-treated cells with 5 μM teriflunomide). **c**, **d** The mean fluorescence intensities obtained were normalized to LPS-stimulated control (*black column*). For comparison of the untreated (0 μM) and teriflunomide-treated samples (0.25–5 μM), a *t* test against the expected mean of one was used (*n* = 4–5). *P* values <0.05 were considered significant. *Asterisks* denote a significant difference versus the LPS-stimulated control (*p < 0.05)

Antigen-presenting properties of microglia are well appreciated. As an efficient antigen-presenting cell, microglia present antigens and provide the crucial co-stimulatory signals required for the reactivation of infiltrating autoreactive T cells. Upon activation, microglia upregulate co-stimulatory molecules such as CD86 on their surface and we tested if teriflunomide interfered with this process. Following the treatment of microglia with LPS in the presence or absence of teriflunomide for 48 and 72 h, cell surface expression of the co-stimulatory molecule CD86 was determined by flow cytometry. As expected, CD86 expression was significantly increased in LPS-treated microglia. No significant effect of different concentrations of teriflunomide on CD86 expression was observed after 48 h treatment (Fig. 5a, c). However, the expression of CD86 was slightly but significantly decreased on LPS-treated microglia cultured in the presence of 5 μM teriflunomide for 72 h (Fig. 5b, d).

### Teriflunomide does not inhibit NF-kB activation

NF-kB is an important transcription factor which regulates the expression of pro-inflammatory genes and is an ideal target for immunomodulatory and anti-inflammatory therapies. Previous studies have shown that 5–10 μM of leflunomide completely blocked NF-kB activation in T cells, myeloid, and epithelial cells [21].

We therefore investigated whether teriflunomide displayed similar inhibitory effects on NF-kB activation in primary microglia. NF-kB is found as a complex with IkBα in the cytoplasm of resting cells. Upon activation IkBα is phosphorylated and subsequently degraded to release NF-kB for nuclear translocation [22–24]. Hence, we followed the kinetics of IkBα degradation, as a measure of NF-kB activation, in response to LPS/IFN-γ in microglia pre-treated with 5 μM teriflunomide for 12 h.

**Fig. 6:**
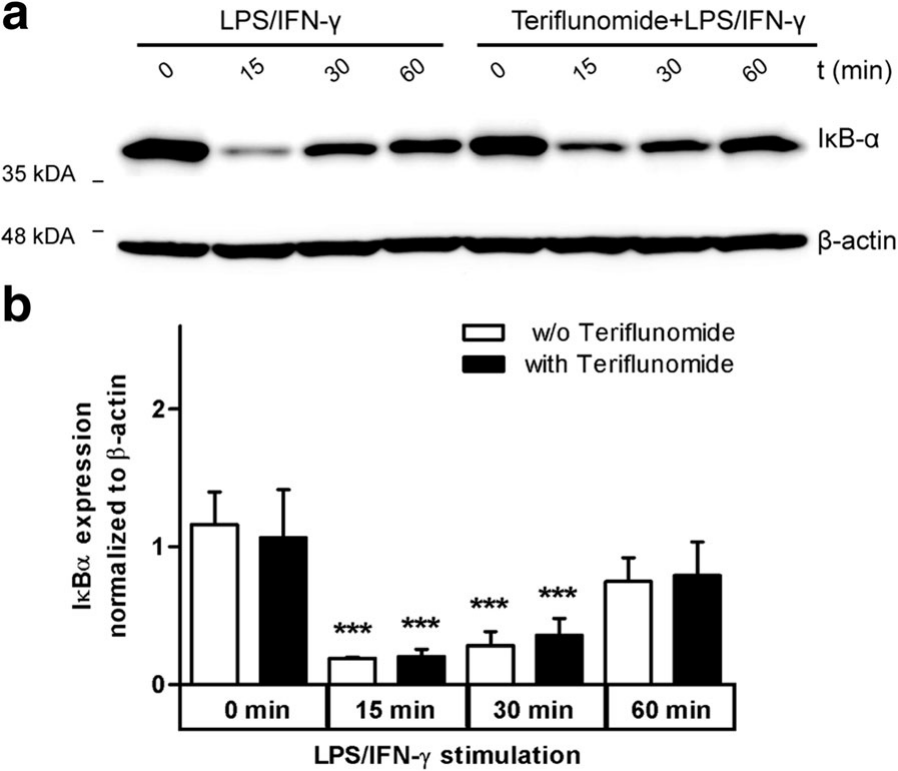
No effect of teriflunomide on the NF-kB signaling pathway. **a** Western blot analysis of teriflunomide pre-treated microglia stimulated with LPS/IFN-γ (100 ng/ml; 50 ng/ml) for the indicated times using IkBα antibody. β-actin was used as loading control. **b** Summary of four independent experiments as in **a** normalized to β-actin and expressed as mean ± SD. Data were analyzed by repeated measures ANOVA with Bonferroni’s multiple comparison *t* test compared to the LPS/IFN-γ control. Significant effects are indicated by *asterisks* (***p < 0.001)

As demonstrated in Fig. 6a, IkBα degradation was evident within 15 min of LPS/IFN-γ-treatment. However, IkBα degradation was unaffected by teriflunomide treatment suggesting that teriflunomide had no impact on this regulation in activated microglia at the physiologically relevant concentration used here (Fig. 6b).

### Phagocytic activity of microglia is unaffected by teriflunomide

**Fig. 7:**
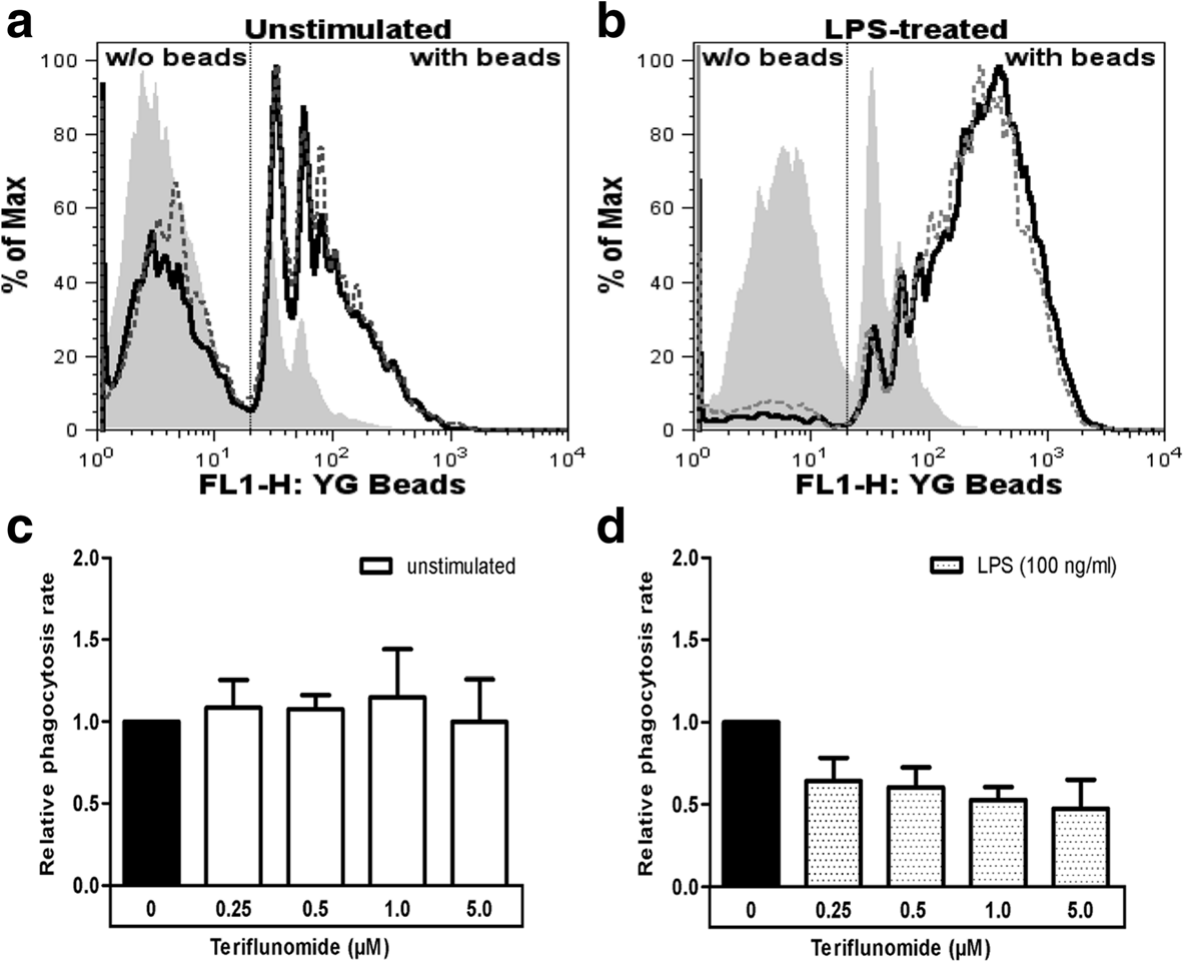
Phagocytic activity of microglia does not change after treatment with teriflunomide. Flow cytometric histograms of unstimulated (**a**) and LPS-treated (**b**) microglia represent the mean fluorescence intensity which displays the amount of incorporated fluorescent latex beads (*filled*: 4 °C control; *solid line*: cells without teriflunomide; *dotted line*: cells with 5 μM teriflunomide). **c**, **d** Data are expressed as the ratio of untreated microglia in relation to teriflunomide-treated microglia. All data are given as mean ± SD of four independent experiments. For comparison to medium control, repeated measures ANOVA with Bonferroni’s multiple comparison *t* tests was used

Phagocytosis is one of the major functions of microglia. Microglia treated with or without LPS were incubated with different concentrations (0.25–5 μM) of teriflunomide for 24 h. Subsequently, the uptake of latex beads by microglia was measured by flow cytometry. As shown in Fig. 7, teriflunomide did not change the phagocytic activity of both untreated and LPS-treated microglia.

## Discussion

In 2012, teriflunomide was approved for the treatment of RRMS in the USA [25]. Teriflunomide selectively inhibits the proliferation of activated T and B lymphocytes. This effect is achieved already at nanomolar concentrations of teriflunomide by blockage of DHODH, an enzyme involved in de novo pyrimidine synthesis [4]. When used at very high concentrations (>50 μM), teriflunomide regulates also other important immune cell functions [26, 27]. For example, treatment with teriflunomide decreased release of pro-inflammatory cytokines in human macrophages or peripheral blood lymphocytes such as IL-6, IL-17, and TNF-α [28–30]. However, pharmacokinetic studies have revealed that only 1–2 % of the peripheral teriflunomide might reach the CNS owing to its low BBB permeability. It is thus conceivable that resident CNS cells such as microglia may be exposed to low teriflunomide concentrations around 0.2–0.6 mg/l in vivo, equivalent to approximately 1–3 μM [10, 11]. In order to evaluate further mechanisms of action of teriflunomide, we investigated its effects on microglia in a range of concentrations between 0.25 and 5 μM that may be reached in the CNS by oral administration.

There is only limited knowledge on how teriflunomide influences the phenotype and function of microglia, the resident immune cell in the CNS. Microglia characteristically undergo rapid proliferation in response to an inflammatory insult in the CNS [31]. From our own experiences and from published reports, we know that in mixed glial cell cultures, microglia rapidly proliferate from days 4–5 and that this process is further enhanced by the addition of growth factors such as GM-CSF [32, 33]. Interestingly, we observed that microglia upregulated DHODH gene expression upon activation. Consequently, we expected similar inhibitory effects of teriflunomide on microglial proliferation. We therefore treated mixed glial cell cultures directly with teriflunomide. Teriflunomide treatment significantly lowered the yield of microglia from GM-CSF-treated mixed glial cell cultures. A reduced frequency of BrdU^+^ microglia indicated decreased proliferation (Fig. 3). However, the exact mechanism how this inhibition is mediated remains to be clarified. We could exclude cytotoxicity influencing our results as we did not detect morphological changes as well as microglial cell death in cells exposed to teriflunomide (Fig. 2; Additional file 1: Figure S2, Additional file 2: Figure S3). Since there was an increased expression of DHODH-mRNA in microglia in response to GM-CSF-treatment (Fig. 1b), we can speculate that the anti-proliferative effect of teriflunomide on activated microglia might be mediated through DHODH inhibition. However, we do not have direct evidence to support this, a DHODH-independent mechanism may also act, as we observed proliferation of microglia in mixed glial cell cultures devoid of GM-CSF is similarly affected by teriflunomide treatment (data not shown), and the concentration required to induce this inhibition is much higher than the concentration that is sufficient to inhibit T cell proliferation (Additional file 3: Figure S1).

Apart from anti-proliferative effects, reports suggest that teriflunomide has significant influence on the inflammatory response of various immune cells. For example, Cutolo et al. showed that leflunomide, the prodrug of teriflunomide, has a concentration-dependent anti-inflammatory effect on cultured synovial macrophages from patients with rheumatoid arthritis [34]. Similar to macrophages, microglia can attain a pro-inflammatory (M1-like) or an anti-inflammatory (M2-1ike) phenotype [35]. This led us to ask if teriflunomide has similar effects on the M1- and M2-like phenotype of microglia in vitro. Interestingly, at 5 μM of teriflunomide, we found a slight but significant enhancement in the mRNA expression of the anti-inflammatory cytokine IL-10 in microglia exposed to LPS/IFN-γ. This is in accordance with the findings of Korn et al. who observed a similar increase in IL-10 expression in LPS-treated microglia that were exposed to teriflunomide [36]. On the other hand, we observed a decreased expression of the co-stimulatory molecule CD86 on LPS-treated microglia that were exposed to 5 μM of teriflunomide. However, no significant effects were detectable on the mRNA expression of pro-inflammatory mediators in M1-microglia. Similarly, the NF-kB pathway, that regulates the expression of pro-inflammatory cytokines, was unaffected in teriflunomide-treated microglia. This stands in contrast to studies in a human T cell line. In this cell line, 5–10 μM of leflunomide were able to block TNF-α-induced NF-kB activation [21, 37]. This may be due to the lower concentrations of teriflunomide used in our studies. Furthermore, microglia seem to be less responsive to teriflunomide as compared to lymphocytes. Taken together, we believe that low concentrations of teriflunomide have no major effects on pro-inflammatory properties of microglia, but higher concentration might possibly enhance their anti-inflammatory properties.

In MS, infiltrating T cells can mediate CNS damage either by directly targeting neurons or by triggering an inflammatory response in microglia. Furthermore microglia respond to CNS damage and remove the damaged cells by phagocytosis. Phagocytosis is one of the main features of microglial activation. In the past, the importance of microglia in the clearance of myelin debris and subsequent repair processes has been demonstrated [38, 39]. However, we did not observe any enhancing or suppressive effects of teriflunomide on phagocytosis in either untreated or LPS-activated microglia.

## Conclusions

In conclusion, we demonstrate here that by and large, only the highest concentration of teriflunomide that microglia may be exposed to in vivo in the CNS has an effect on proliferation. Furthermore, only stimulation with the highest concentration of teriflunomide (5 μM) had a significant effect on mRNA expression of IL-10. Lower concentrations were not sufficient to modulate significantly further anti- or pro-inflammatory responses. Thus, we provide evidence that teriflunomide may have an effect on microglial proliferation which may be of importance in the treatment of neuroinflammatory CNS diseases. Further in vivo studies are required to corroborate this effect.

## Additional files

Additional file 1: Figure S2. Teriflunomide does not affect cell viability of primary microglia. To investigate possible cytotoxic effects of the drug in the concentrations used in this study, we employed the Alamar blue cell viability assay (Invitrogen, Darmstadt, Germany), which measures the oxidation status of the cells without affecting the function of the electron transport chain. Cells were treated with teriflunomide (stock: 10 mM; dissolved in dimethyl sulfoxide (DMSO, vehicle); 0.25–5 μM) for (A) 24 h, (B) 48 h, or (C) 72 h. The medium was completely changed and replaced with 100 μl cell culture medium supplied with 10 % Alamar blue solution, and cells were further incubated for 4 h. Fluorescence intensity of Alamar blue was measured at 570 nm with a microplate reader (Tecan Sunrise, Crailsheim, Germany). Duplicate measurements were averaged in four independent experiments. Data are normalized to untreated control (1st column), presented as mean ± SD and compared to the untreated control. (TIF 780 kb) Additional file 2: Figure S3. Teriflunomide does not affect cell death in cultured primary microglia. Isolated microglia were treated with LPS (100 ng/ml) and different concentrations of teriflunomide (0.25–5 μM) for 12, 48, or 72 h followed by staining with propidium iodide (PI). (A, B, C) The percentage of gated PI^+^ CD11b/c^+^ cells is presented as mean + SD (*n* = 4). (TIF 718 kb) Additional file 3: Figure S1. Teriflunomide inhibits rodent T cell proliferation. Single-cell suspensions from spleens of adult Sprague-Dawley rats (Crl:CD) were prepared in complete IMDM medium. Freshly isolated rat CD4^+^ T cells were labeled with 2.5 μM CFSE, stimulated with plate-bound anti-CD3/CD28 mAb and treated with different concentrations of teriflunomide (0.25–5 μM) for 65–72 h. Control presents unstimulated CFSE-labeled cells. (A) Total number of T cells as shown in histograms for 0.5 μM teriflunomide was determined by flow cytometry. (B) For comparison of proliferation, the number of proliferating T cells (*n* = 4) repeated measures ANOVA with Bonferroni’s multiple comparison *t* test were used. Significant effects are indicated by asterisks (****p* < 0.001). (TIF 162 kb) Additional file 4: Figure S4. Teriflunomide does not interfere with astrocytic proliferation or secretion of cytokines and growth factors. (A) Astrocytes were treated without GM-CSF in mixed glial cell cultures for 48 h. Cultures were incubated with 10 μM BrdU for the last 16 h, and dividing astrocytes were then visualized by labeling with a FITC-conjugated anti-BrdU antibody. For comparison of untreated (0 μM) and teriflunomide-treated (5 μM) sample (*n* = 3), a paired *t* test was used. (B–E) The columns represent the relative quantity of gene expression of teriflunomide-treated astrocytes following stimulation with GM-CSF for 12 h (dotted columns) or unstimulated (white columns). The dashed line represents the basal expression level in untreated, unstimulated astrocytes. Results are presented as the fold-change of astrocyte-secreted factors normalized to the expression of the reference gene *Hprt1* and were calculated relative to unstimulated, untreated cells. Data from four experiments are represented as the mean ± standard deviation. **p* < 0.05, ***p* < 0.005, ****p* < 0.001, compared with the basal expression level (black column). GM-CSF - granulocyte macrophage-colony stimulating factor; FGF-2 - fibroblast growth factor 2; RQ - relative quantity. (TIF 930 kb)

## Abbreviations

APC: Allophycocyanin
Arg: Arginase
BBB: Blood-brain barrier
BrdU: 5-Bromo-2′-deoxyuridine
cDNA: Complementary deoxyribonucleic acid
CFSE: Carboxyfluorescein succinimidyl ester
CNS: Central nervous system
DHODH: Dihydroorotate dehydrogenase
DMEM: Dulbecco’s Modified Eagle Medium
DMSO: Dimethyl sulfoxide
DMT: Disease-modifying therapy
ECL: Enhanced chemiluminescence
EDTA: Ethylenediaminetetraacetic acid
FCS: Fetal calf serum
FITC: Fluorescein isothiocyanate
GM-CSF: Granulocyte macrophage-colony stimulating factor
HPRT: Hypoxanthine-guanine phosphoribosyl-transferase
HRP: Horseradish peroxidase
IFN: Interferon
IGF-1: Insulin-like growth factor-1
IkBα: Inhibitor of nuclear factor-kB
IL: Interleukin
IMDM: Iscove’s Modified Dulbecco’s Medium
iNOS: Inducible nitric oxide synthase
LPS: Lipopolysaccharide
MFI: Mean fluorescence intensity
MGP: Mixed glial cell preparation
MS: Multiple sclerosis
NF-kB: Nuclear factor-kB
NH_4_Cl: Ammonium chloride
PE: Phycoerythrin
RA: Rheumatoid arthritis
RIPA: Radioimmunoprecipitation assay buffer
RRMS: Relapsing-remitting multiple sclerosis
RT-PCR: Reverse transcription polymerase chain reaction
SD: Standard deviation
TNF: Tumor necrosis factor
