# Supplementary figures and images for: In vitro evaluation of physiologically relevant concentrations of teriflunomide on activation and proliferation of primary rodent microglia
Source: J Neuroinflammation. 2016 Sep 22;13:250. doi: 10.1186/s12974-016-0715-3 (PMC5034581; doi:10.1186/s12974-016-0715-3)

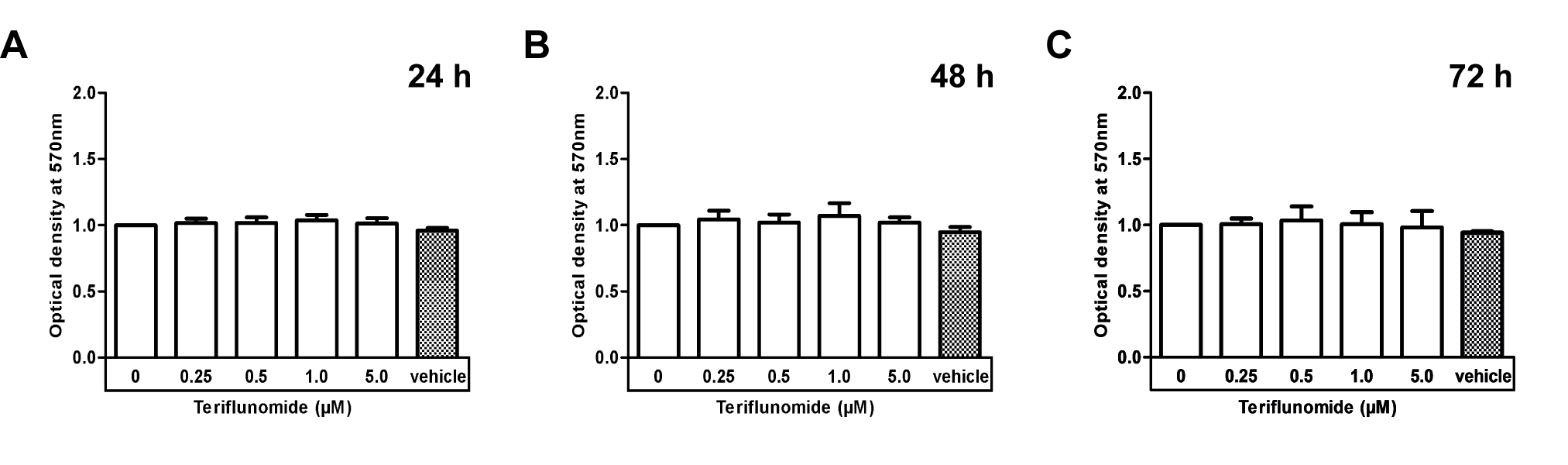

Supplement: Additional file 1: Figure S2. — Teriflunomide does not affect cell viability of primary microglia. To investigate possible cytotoxic effects of the drug in the concentrations used in this study, we employed the Alamar blue cell viability assay (Invitrogen, Darmstadt, Germany), which measures the oxidation status of the cells without affecting the function of the electron transport chain. Cells were treated with teriflunomide (stock: 10 mM; dissolved in dimethyl sulfoxide (DMSO, vehicle); 0.25–5 μM) for (A) 24 h, (B) 48 h, or (C) 72 h. The medium was completely changed and replaced with 100 μl cell culture medium supplied with 10 % Alamar blue solution, and cells were further incubated for 4 h. Fluorescence intensity of Alamar blue was measured at 570 nm with a microplate reader (Tecan Sunrise, Crailsheim, Germany). Duplicate measurements were averaged in four independent experiments. Data are normalized to untreated control (1st column), presented as mean ± SD and compared to the untreated control. (TIF 780 kb) [file 12974_2016_715_MOESM1_ESM.tif]

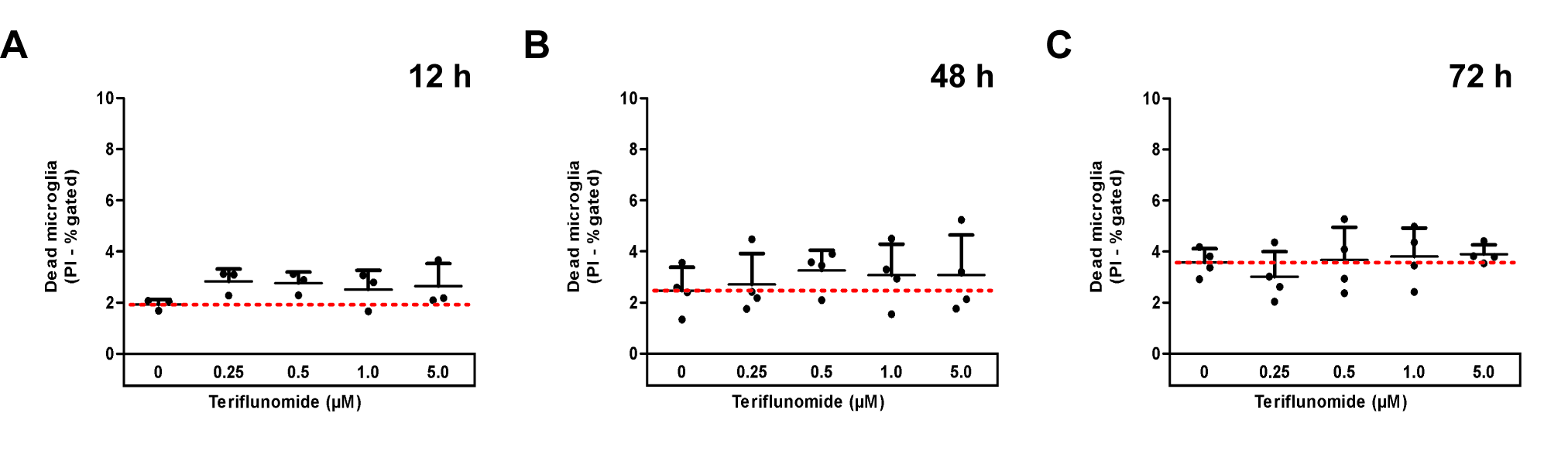

Supplement: Additional file 2: Figure S3. — Teriflunomide does not affect cell death in cultured primary microglia. Isolated microglia were treated with LPS (100 ng/ml) and different concentrations of teriflunomide (0.25–5 μM) for 12, 48, or 72 h followed by staining with propidium iodide (PI). (A, B, C) The percentage of gated PI+ CD11b/c+ cells is presented as mean + SD (n = 4). (TIF 718 kb) [file 12974_2016_715_MOESM2_ESM.tif]

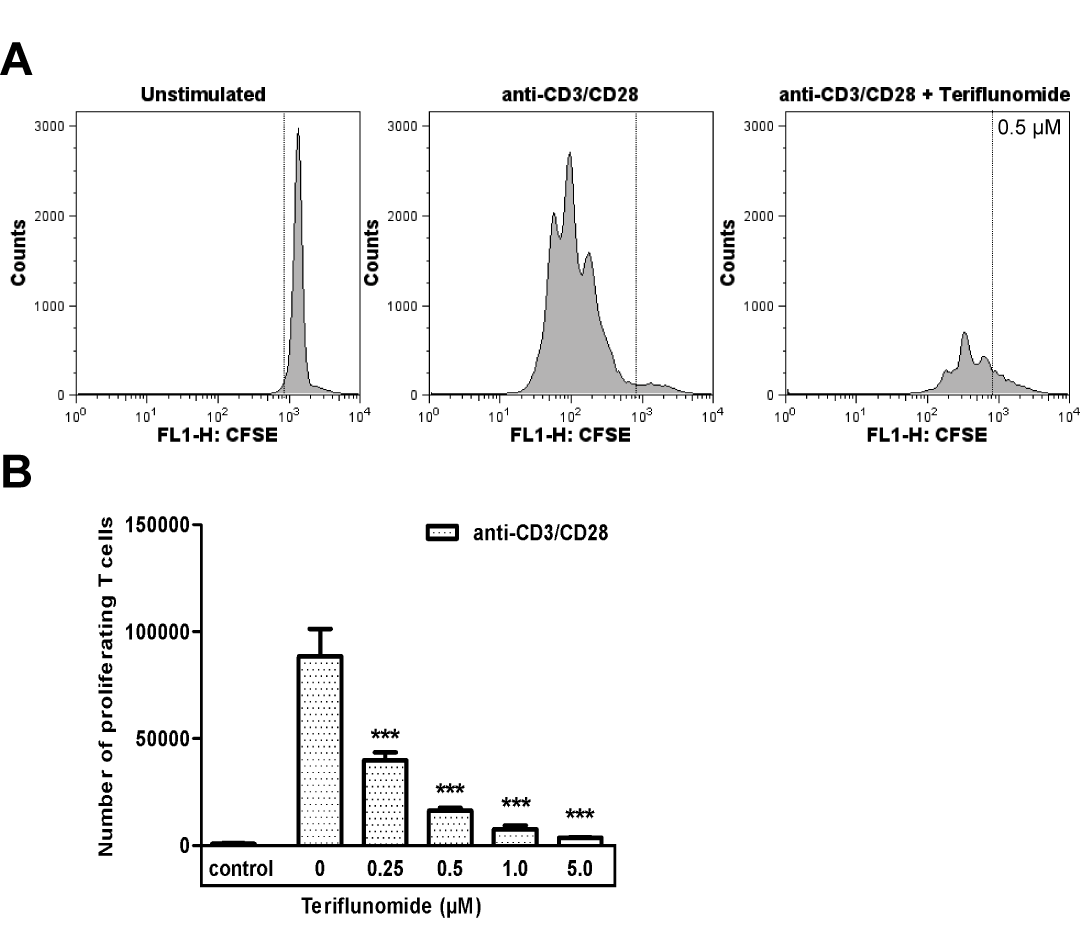

Supplement: Additional file 3: Figure S1. — Teriflunomide inhibits rodent T cell proliferation. Single-cell suspensions from spleens of adult Sprague-Dawley rats (Crl:CD) were prepared in complete IMDM medium. Freshly isolated rat CD4+ T cells were labeled with 2.5 μM CFSE, stimulated with plate-bound anti-CD3/CD28 mAb and treated with different concentrations of teriflunomide (0.25–5 μM) for 65–72 h. Control presents unstimulated CFSE-labeled cells. (A) Total number of T cells as shown in histograms for 0.5 μM teriflunomide was determined by flow cytometry. (B) For comparison of proliferation, the number of proliferating T cells (n = 4) repeated measures ANOVA with Bonferroni’s multiple comparison t test were used. Significant effects are indicated by asterisks (***p < 0.001). (TIF 162 kb) [file 12974_2016_715_MOESM3_ESM.tif]

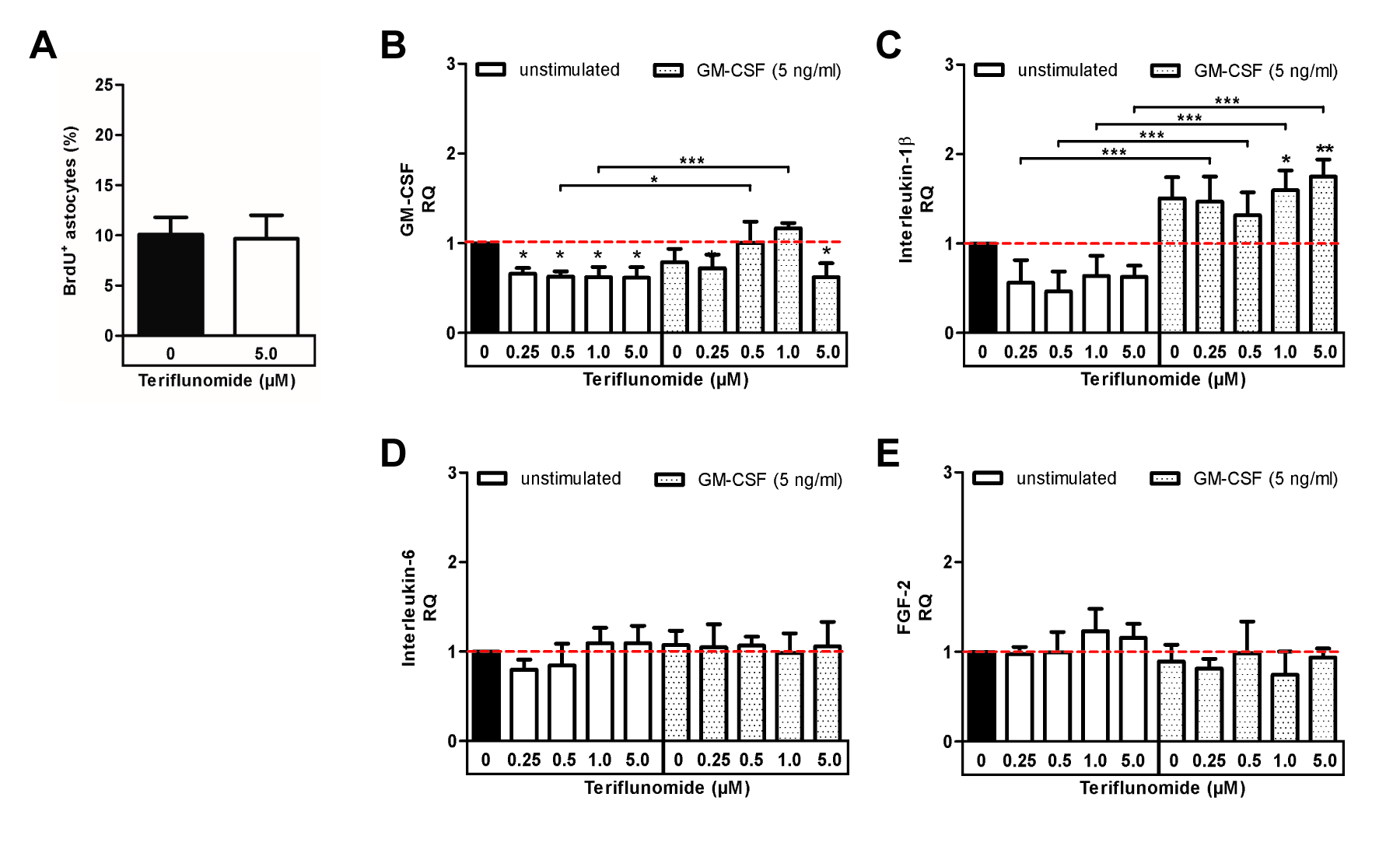

Supplement: Additional file 4: Figure S4. — Teriflunomide does not interfere with astrocytic proliferation or secretion of cytokines and growth factors. (A) Astrocytes were treated without GM-CSF in mixed glial cell cultures for 48 h. Cultures were incubated with 10 μM BrdU for the last 16 h, and dividing astrocytes were then visualized by labeling with a FITC-conjugated anti-BrdU antibody. For comparison of untreated (0 μM) and teriflunomide-treated (5 μM) sample (n = 3), a paired t test was used. (B–E) The columns represent the relative quantity of gene expression of teriflunomide-treated astrocytes following stimulation with GM-CSF for 12 h (dotted columns) or unstimulated (white columns). The dashed line represents the basal expression level in untreated, unstimulated astrocytes. Results are presented as the fold-change of astrocyte-secreted factors normalized to the expression of the reference gene Hprt1 and were calculated relative to unstimulated, untreated cells. Data from four experiments are represented as the mean ± standard deviation. *p < 0.05, **p < 0.005, ***p < 0.001, compared with the basal expression level (black column). GM-CSF - granulocyte macrophage-colony stimulating factor; FGF-2 - fibroblast growth factor 2; RQ - relative quantity. (TIF 930 kb) [file 12974_2016_715_MOESM4_ESM.tif]
